# Synthesis and Characterization of a Novel Biocompatible Alloy, Ti-Nb-Zr-Ta-Sn

**DOI:** 10.3390/ijms221910611

**Published:** 2021-09-30

**Authors:** Yuliya Y. Khrunyk, Sabrina Ehnert, Stella V. Grib, Anatoly G. Illarionov, Stepan I. Stepanov, Artemiy A. Popov, Maxim A. Ryzhkov, Sergey V. Belikov, Zeqian Xu, Frank Rupp, Andreas K. Nüssler

**Affiliations:** 1 Department of Heat Treatment and Physics of Metal, Ural Federal University, Mira Str. 19, 620002 Yekaterinburg, Russia; s.v.grib@urfu.ru (S.V.G.); a.g.illarionov@urfu.ru (A.G.I.); s.i.stepanov@urfu.ru (S.I.S.); a.a.popov@urfu.ru (A.A.P.); m.a.ryzhkov@urfu.ru (M.A.R.); s.v.belikov@mail.ru (S.V.B.); 2 Siegfried Weller Institute for Trauma Research, BG Unfallklinik Tübingen, Eberhard Karls Universität Tübingen, Schnarrenbergstr. 95, 72076 Tübingen, Germany; sabrina.ehnert@med.uni-tuebingen.de; 3 Section Medical Material Science and Technology, University Hospital Tübingen, Osianderstr. 2-8, 72076 Tübingen, Germany; xuzeqian934130@gmail.com (Z.X.); Frank.Rupp@med.uni-tuebingen.de (F.R.); 4 Department of Prosthodontics, Shanghai Ninth People’s Hospital, Shanghai Jiao Tong University School of Medicine, Shanghai 200011, China; 5 National Center for Stomatology, Shanghai Key Laboratory of Stomatology, College of Stomatology, Shanghai Jiao Tong University, Shanghai 200011, China; 6 National Clinical Research Center for Oral Diseases, Shanghai Jiao Tong University, Shanghai 200011, China; 7 Shanghai Engineering Research Center of Advanced Dental Technology and Materials, Shanghai Jiao Tong University, No. 639 Zhizaoju Road, Shanghai 200011, China

**Keywords:** biomedical implants, low-modulus Ti alloys, O_2_ plasma treatment, tensile properties, biocompatibility

## Abstract

Many current-generation biomedical implants are fabricated from the Ti-6Al-4V alloy because it has many attractive properties, such as low density and biocompatibility. However, the elastic modulus of this alloy is much larger than that of the surrounding bone, leading to bone resorption and, eventually, implant failure. In the present study, we synthesized and performed a detailed analysis of a novel low elastic modulus Ti-based alloy (Ti-28Nb-5Zr-2Ta-2Sn (TNZTS alloy)) using a variety of methods, including scanning electron microscopy, transmission electron microscopy, X-ray diffraction, and tensile test. Additionally, the in vitro biocompatibility of the TNZTS alloy was evaluated using SCP-1, SaOs-2, and THP-1 cell lines and primary human osteoblasts. Compared to Ti-6Al-4V, the elastic modulus of TNZTS alloy was significantly lower, while measures of its in vitro biocompatibility are comparable. O_2_ plasma treatment of the surface of the alloy significantly increased its hydrophilicity and, hence, its in vitro biocompatibility. TNZTS alloy specimens did not induce the release of cytokines by macrophages, indicating that such scaffolds would not trigger inflammatory responses. The present results suggest that the TNZTS alloy may have potential as an alternative to Ti-6Al-4V.

## 1. Introduction

Research addressing titanium-based biocompatible alloys has captured great interest in recent decades [1,2,3,4,5,6,7,8,9,10]. Alloying and specific treatments, including thermal and deformation processing, results in reduced elastic modulus (or Young’s modulus) close to that of bone (10–30 GPa) [1,2,11]. Indeed, the production of low elastic modulus alloys is crucial for implantology, where implant failure is often associated with a stiffness mismatch between the implanted material and human bone [12,13].

Ti-6Al-4V alloy is widely used to fabricate biomedical implants, such as dental posts and parts of total joint replacements [1,2,3,4,11,12,13], because this alloy has many attractive properties, such as the abundance of Ti in nature, biocompatibility [14], low density, and a high strength-to-weight ratio [15,16]. However, the elastic modulus of this alloy (116 GPa [17]), which has a hexagonal crystal structure, is several orders of magnitude higher than that of the surrounding human cancellous bone (0.01–3 GPa [1,2,11]). This phenomenon, known as stress shielding, has been identified as causing bone resorption, which eventually leads or contributes to implant failure. A decrease in elastic modulus of Ti-based alloys can be achieved by alloying, thermal treatment, and deformation processing. Alloying stabilizes high-temperature modification of the Ti body-centered cubic (bcc) β-phase with low elastic modulus [15], replacing low-temperature α-modification with the hexagonal close-packed lattice structure. Currently, isomorphic β-stabilizers (Nb and Ta) are the most frequently used alloying elements [18,19,20] (Table 1). Other β-stabilizing alloying elements, both isomorphic ones (Mo, V) and eutectoid-forming ones (Fe, Ni, Cr, Mn, Si, Co, Cu), have limited or no biocompatibility [14,21], which rules them out for use in low elastic modulus Ti-based alloys intended for implant applications. Further reduction of elastic modulus of Ti-based alloys that contain Nb and Ta can be achieved by incorporation of the so-called neutral titanium strengtheners Zr and Sn [15], both of which are biocompatible elements [14,21] (Table 1).

The biocompatibility of Ti-Nb-based alloys has been shown in different studies [4,14,22]. In particular, using cell cultures, good biocompatibility was recorded for TiNbTa [23], Ti-40Nb [24], Ti-Nb [25,26,27], and Nb-Ti-Zr [28].

**Figure 1:**
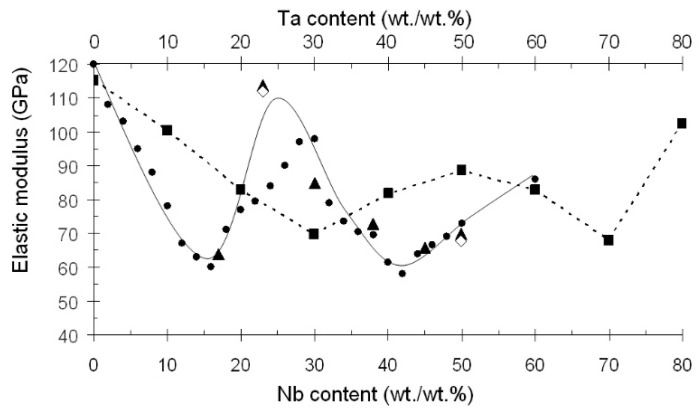
Variation of elastic modulus of Ti-based alloys with their Nb and Ta contents. ●—Ti-Nb [33]; ▲—Ti-Nb [18]; ◊—Ti-Nb [34]; ■—Ti-Ta [20].

The elastic modulus of alloys in the Ti-Nb system is lower than those in the Ti-Ta system (Figure 1) [18,20]. Notably, low elastic modulus (about 60 GPa) in Ti-Nb alloys is achieved when the Nb content is 40–42 wt./wt.% [18]. If a molybdenum equivalent ([Mo]eq) is used to characterize the stability of β-hard solution to transformations, the following formula is employed [32]: [Mo]eq = [Mo] + 0.2[Ta] + 0.28[Nb] + 0.4[W] + 0.67[V] + 1.25[Cr] +1.25[Ni] + 1.7[Mn] +1.7[Co] +2.5[Fe] (wt./wt.%); then, for Ti-(40–42) wt% Nb alloys, [Mo]eq = 11.2–11.8.

Our research aimed to design, synthesize, and characterize a novel alloy, Ti-38Nb-5Zr-2Ta-2Sn (TNZTS alloy), which has a comparable molybdenum equivalent ([Mo]eq = 11.0) to Ti-29Nb-13Ta-4.6Zr while being less expensive. Characterization of the novel alloy included determination of microstructure, morphology, phase composition, and a large collection of properties relevant to its potential use as an alternative to Ti-6Al-4V alloys, such as elastic modulus, hydrophilicity, and biocompatibility.

## 2. Results

### 2.1. Microstructure and Composition of the Cast NTZTS

**Figure 2:**
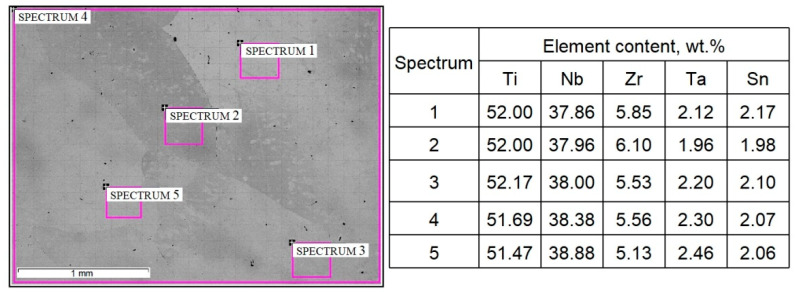
Microstructure and composition of as-cast TNZTS alloy, with highlighted areas pointing to the sites of the EDX analysis. Scale bar = 1 mm.

**Figure 3:**
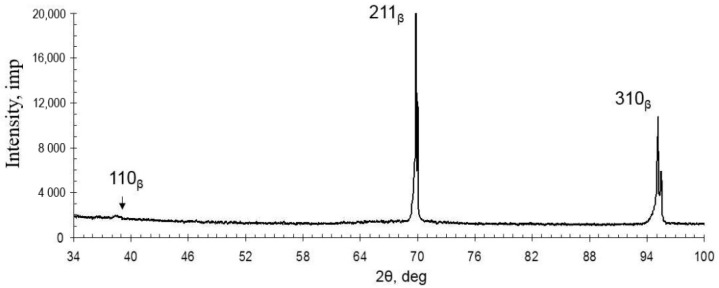
Diffraction pattern of the as-cast TNZTS alloy.

According to SEM and XRD (Figure 2 and Figure 3) data, the as-cast TNZTS alloy has a single-phase β-structure.

The structure of TNZTS alloy comprises large β-grains 1 mm in size (Figure 2). The X-ray diffraction pattern obtained from the surface of the as-cast alloy showed X-ray diffraction peaks only corresponding to the β-phase (Figure 3). 

The EDX results (Figure 2) show that the chemical composition of the as-cast TNZTS alloy, rounded to 0.1 wt./wt.%, is as follows: Ti-38.4Nb-5.6Zr–2.3Ta-2.1Sn. In the as-cast alloy, liquation from one β-grain to another was observed in the range of 1% for Nb, 0.5% for Zr and Ta, and 0.2% for Sn (spectra 2–5, Figure 2). Hence, the as-cast TNZTS alloy is characterized as having a large-grained structure of non-homogenous β-solid solution.

### 2.2. Microstructure of the Forged TNZTS Alloy

**Figure 4:**
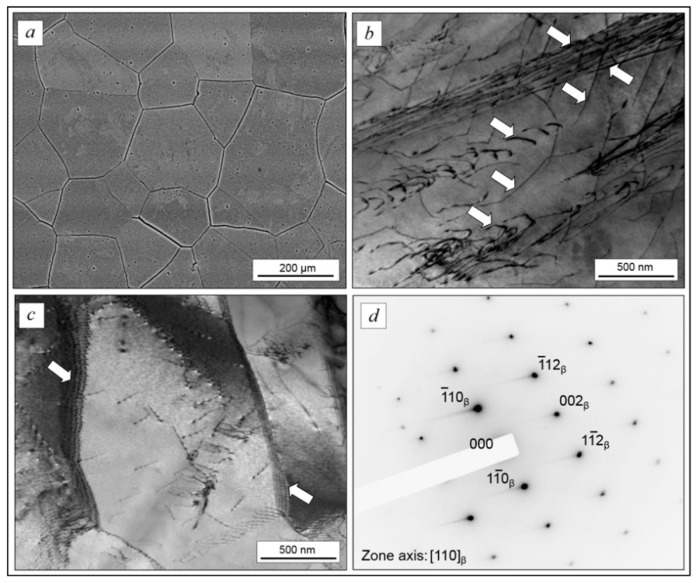
The microstructure of forged TNZTS alloy rod: polyhedral β-grains (**a**); dislocation structure, dislocations are indicated by arrows (**b**); and sub-grains structure (**c**) electron diffraction pattern from the β-matrix (**d**). Images were obtained in the transverse cross-section of the rod. Scale bar = 200 μm (**a**), 500 nm (**b**,**c**).

**Figure 5:**
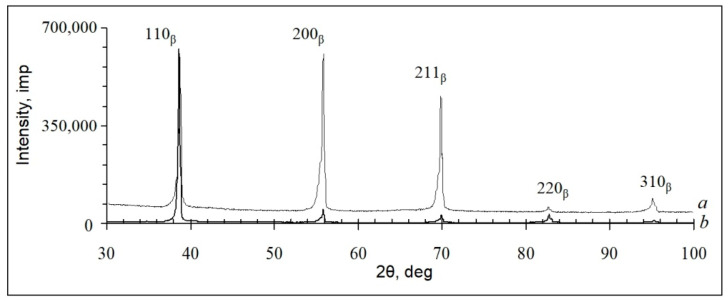
Diffraction patterns of forged TNZTS rod taken from longitudinal cross-section (a) and transverse cross-section (b).

The TEM, SEM, and XRD results (Figure 4 and Figure 5) show that the forged TNZTS alloy also has a single β-phase structure. TEM of the forged rod (Figure 4b–d) indicated the presence of both separated and aggregated dislocations within the β-matrix (Figure 4b). β-grains can be split into separate sub-grains with an increased density of dislocations at sub-boundaries (Figure 4c). The electron diffraction patterns demonstrate the reflexes of the only metastable β-solid solution (Figure 4d).

Analysis of the intensity of the peaks in the diffraction pattern taken from the transverse and longitudinal cross-sections indicated the presence of crystallographic texture in the forged rod (Figure 5). Diffraction peaks {200} and {112} have higher intensity in the longitudinal cross-section compared to a textureless state, whereas the {110} peak has the maximum intensity in the transverse cross-section. Thus, the <110> direction, being perpendicular to the {110} plane, lies in the {200} and {112} planes. Therefore, a mixed {001} <110> and {112} <110> texture developed in the longitudinal cross-section of the forged bar, which is typical for the rolling texture of bcc metals [35].

**Figure 6:**
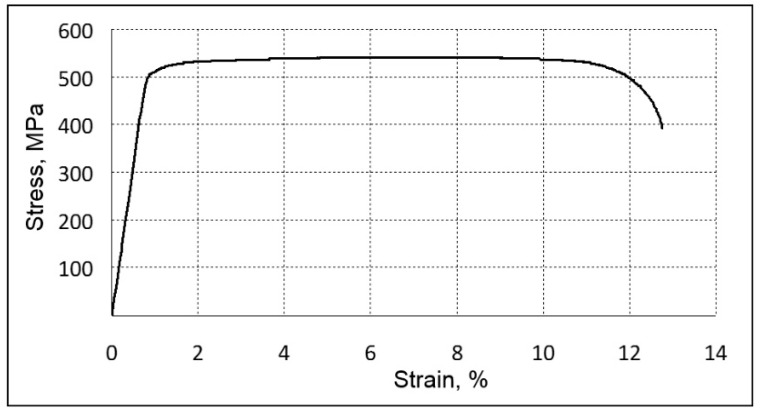
Sample tensile stress–strain results (forged TNTZS alloy specimen).

A sample tensile stress–strain curve obtained using a forged TNZTS alloy specimen is shown in Figure 6. A summary of tensile properties of the alloy (obtained from the tensile test results) as well as its hardness and other measures of stiffness, obtained using different methods, is presented in Table 2.

The analysis of the tensile test results indicated the absence of intense strain hardening in the TNZTS alloy (YS and UTS are within 5% of each other). The alloy, though having relatively low strength and hardness, showed high ductility (Table 2). This was due to the formation of a one-phase metastable state with a bcc crystal structure having a large number of sliding systems [36] and a relatively low density of dislocations within a coarse β-grain (Figure 4).

Elastic modulus obtained from various tests, namely, the tensile test (E), DMA (E*), and microindentation (Er), yielded similar values in the range of 60–63 GPa (Table 2).

### 2.3. Cell Attachment 

**Figure 7:**
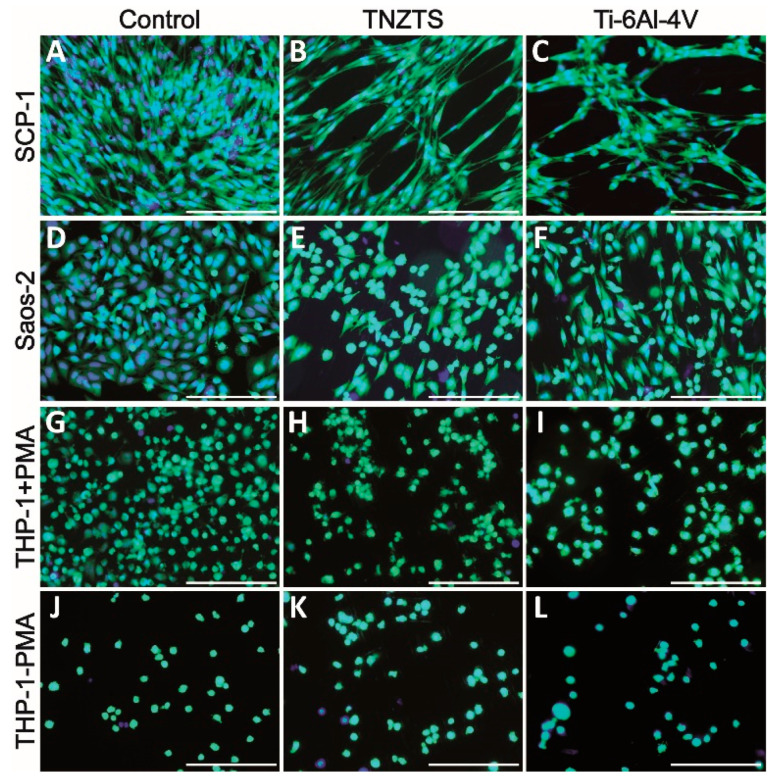
Cell viability on cell culture plate plastic (control, (**A**,**D**,**G**,**J**)) and tested metallic scaffolds (TNZTS, (**B**,**E**,**H**,**K**); Ti-6Al-4V, (**C**,**F**,**I**,**L**)). SCP-1, Saos-2, and THP-1 (with and without PMA) were cultured on analyzed substrates for 24 h. Viable cells exposed to Calcein-AM exhibited green fluorescence. Nuclei (blue fluorescence) were labeled with Hoechst 33342 counterstain. Sb: 200 µm.

**Figure 8:**
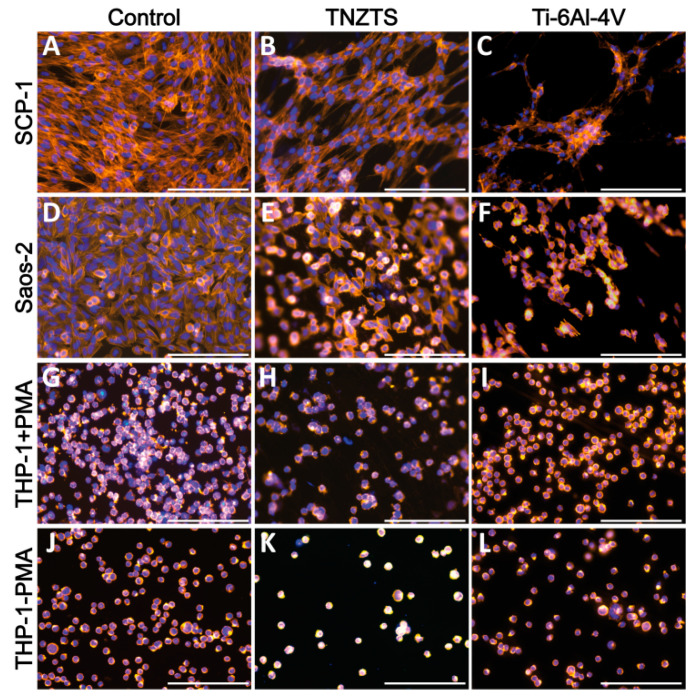
Cell adhesion on cell culture plate plastic (control, (**A**,**D**,**G**,**J**)) and tested metallic scaffolds (TNZTS, (**B**,**E**,**H**,**K**); Ti-6Al-4V, (**C**,**F**,**I**,**L**)). SCP-1, Saos-2, and THP-1 (with and without PMA) were cultured on analyzed substrates for 24 h. Cell actin and nuclei were stained by Alexa Fluor 555 Phalloidin (orange) and Hoechst 33342 (blue), respectively. Sb: 200 µm.

After 24 h of cultivation, cell viability was visualized by Calcein-AM staining (Figure 7). Both SCP-1 and SaOs-2 cells displayed a higher density of viable cells on cell culture plate plastic surfaces (Figure 7A,D) compared to metallic scaffolds (Figure 7B,C,E,F). The cell density of the osteogenic (precursor-) cells was slightly higher on the TNZTS alloy surface than on the Ti-6Al-4V surface (Figure 7B,C,E,F,H,I,K,L). This finding was amplified by staining for the cytoskeleton (Figure 8), which showed better attachment of the osteogenic cells to the TNZTS alloy surface than to the Ti-6Al-4V surface (Figure 8B,C,E,F). Regarding monocytic THP-1 cells, the TNZTS alloy and Ti-6Al-4V scaffolds did not induce any activation of cells when compared to cell culture plate plastic (Figure 7J,K,L; Figure 8J,K,L). THP-1 cells attached better to the Ti-6Al-4V surface than the TNZTS alloy surface, with the difference being more pronounced when THP-1 cells were activated with PMA (Figure 8H,I,K,L).

### 2.4. Release of Neutrophil Extracellular Traps (NETs)

**Figure 9:**
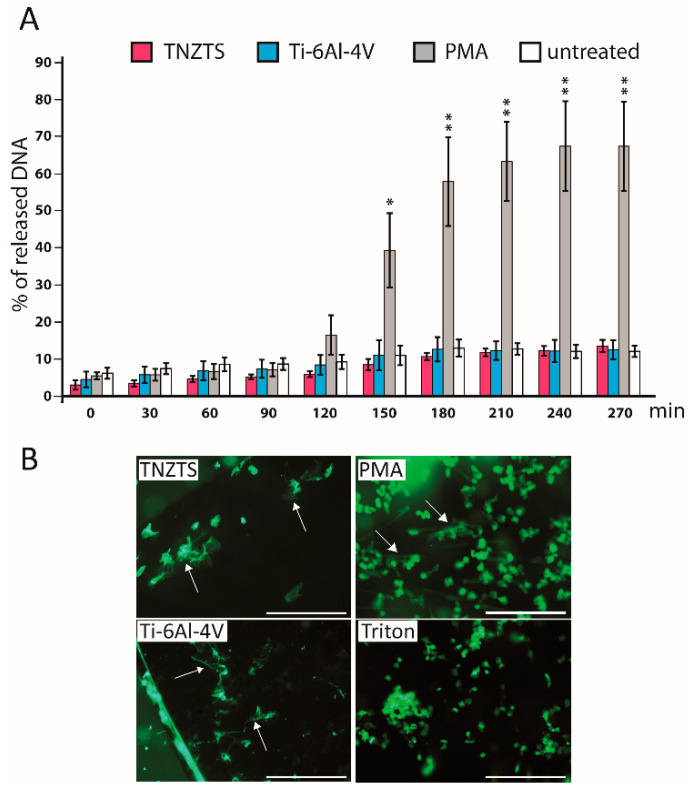
(**A**). Sytox Green Assay to determine NETosis rate in the presence of metallic scaffolds. Fluorescence signal was detected every 30 min up to 4.5 h. Fluorescence signals were recorded for untreated neutrophils and neutrophils cultured with TNZTS, Ti, and PMA (positive control). The data are presented as % of DNA released with Triton-treated, 100% lysed neutrophils. Data represent mean ± SD, *n* = 3. Asterisks show level of statistical significance (*p*) for untreated cells and cells incubated on metallic scaffolds (TNZTS alloy and Ti-6Al-4V) versus PMA-induced neutrophils: * for *p* < 0.1; ** for *p* < 0.05. (**B**). Microscopic visualization of neutrophil extracellular traps. NETs released by neutrophils (marked by arrows) were visualized after 150 min incubation onto TNZTS or Ti and subsequent stimulation with PMA (100 nM). Then, cell necrosis was induced by 0.5% Triton X-100. Sb: 400 µm. The images shown are a representative dataset of four independent experiments.

Neutrophils stimulated with PMA demonstrated an increase in fluorescence at 90 min that grew steadily, reaching a plateau at 210 min (Figure 9A). At 150 min, the cells were also analyzed by fluorescence microscopy (Figure 9B). Although some NET-like structures were detected on both Ti-6Al-4V and TNZTS alloy scaffolds, fluorescence recorded for neutrophils cultured on these surfaces did not differ from the signal shown for untreated cells (Figure 9A), indicating that only a very small amount of NETs was released.

### 2.5. Mitochondrial Activity of Cells

**Figure 10:**
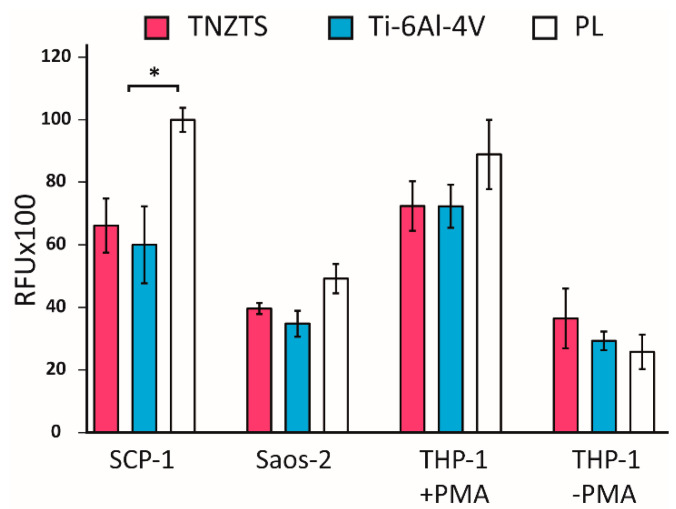
Summary of the results of resazurin reduction in cell cultures: SCP-1, Saos-2, THP-1 (with and without PMA) at 24 h after seeding indicating mitochondrial activity. Tested cells were cultured on metallic scaffolds (TNZTS alloy and Ti-6Al-4V) and cell culture plate plastic (PL). The measurements were taken at 150 min following incubation with resazurin; * for *p* < 0.1; data represent mean ± SD; *n* = 3.

After 24 h of cultivation, the mitochondrial activities of cells seeded on the tested substrates were analyzed by resazurin conversion to resorufin (Figure 10). These data were further confirmed by protein content analysis (Figure S1). In line with viability staining, resazurin conversion did not differ significantly between two metallic substrates for all tested cells.

### 2.6. O_2_ Plasma Treatment and Hydrophilicity

**Figure 11:**
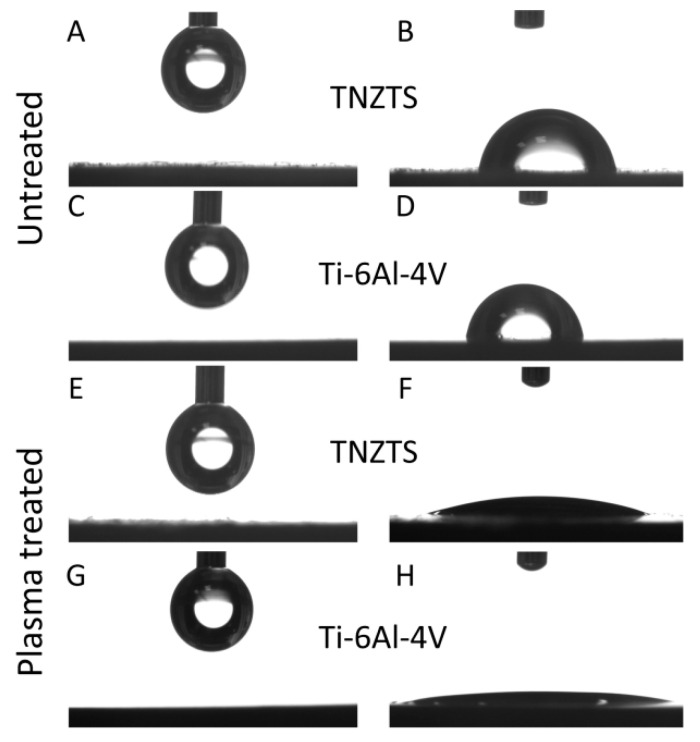
Schematic presentation of the sessile drop contact angle test results. A drop prior to the application on untreated TNZTS (**A**) and Ti-6Al-4V (**C**) and after being set from the needle onto untreated TNZTS (**B**) and Ti-6Al-4V (**D**). Following O_2_ plasma treatment of TNZTS and Ti-6Al-4V, wettability analysis pointed to increased hydrophilicity. Drops (before the application on TNZTS (**E**) and Ti-6Al-4V (**G**)) were almost completely infiltrated into the specimen (Ti-6Al-4V, (**H**)) or displayed a significant reduction in their contact angle (TNZTS, (**F**)).

It is known that surface hydrophilicity plays an important role in the biocompatibility of materials [40,41]. Both the TNZTS alloy and Ti-6Al-4V displayed low hydrophilicity, having contact angles of 69.4 ± 8.7° and 78.4 ± 6.2° (Figure 11A–D). This is likely the reason why fewer cells attached to the metal surfaces in comparison to the cell culture plastic surface. To improve the biocompatible properties of the alloy, O_2_ plasma treatment was applied to the alloy surfaces. This resulted in a significant increase in their hydrophilicity, with contact angles of O_2_-plasma-treated TNZTS alloy and Ti-6Al-4V surfaces being 10.03 ± 2.11° and 6.33 ± 3.28°, respectively (Figure 11E–H).

### 2.7. Cell Response to O_2_-Plasma-Treated Metallic Scaffolds

**Figure 12:**
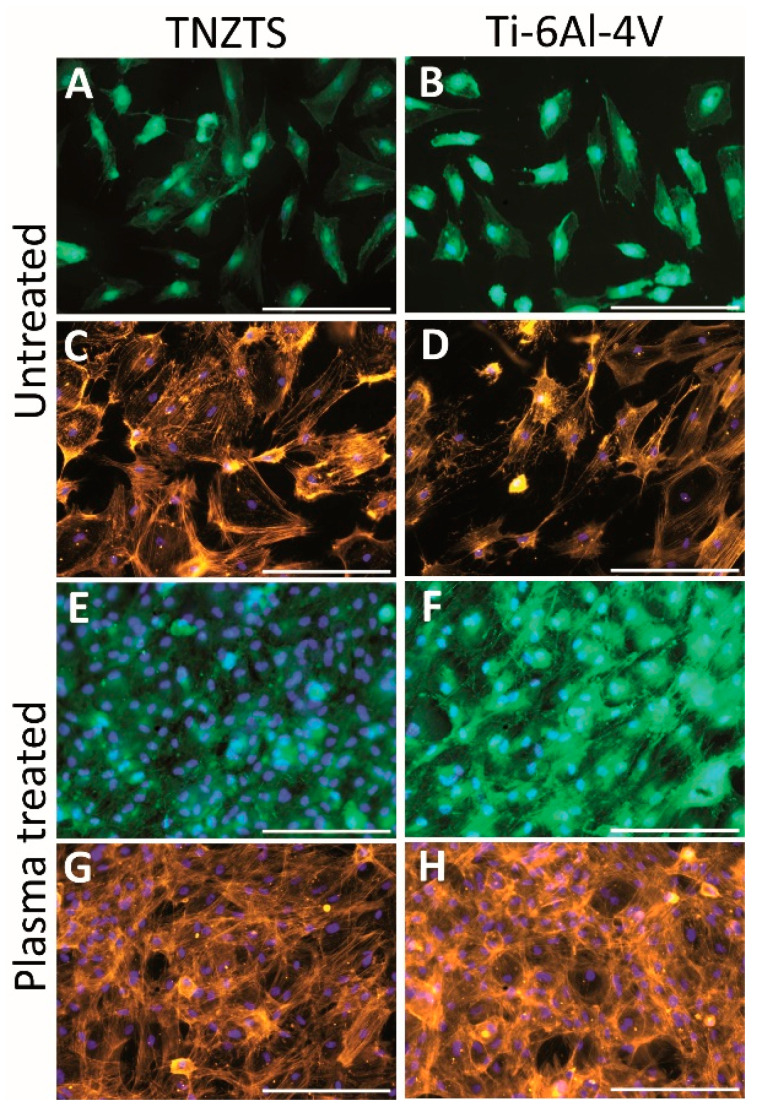
Summary of the cell viability (**A**,**B**,**E**,**F**) and cell adhesion (**C**,**D**,**G**,**H**) results on untreated and O_2_-plasma-treated metallic scaffolds (TNZTS alloy, Ti-6Al-4V). Primary human osteoblasts were cultured on analyzed substrates for 24 h. Sb: 200 µm.

**Figure 13:**
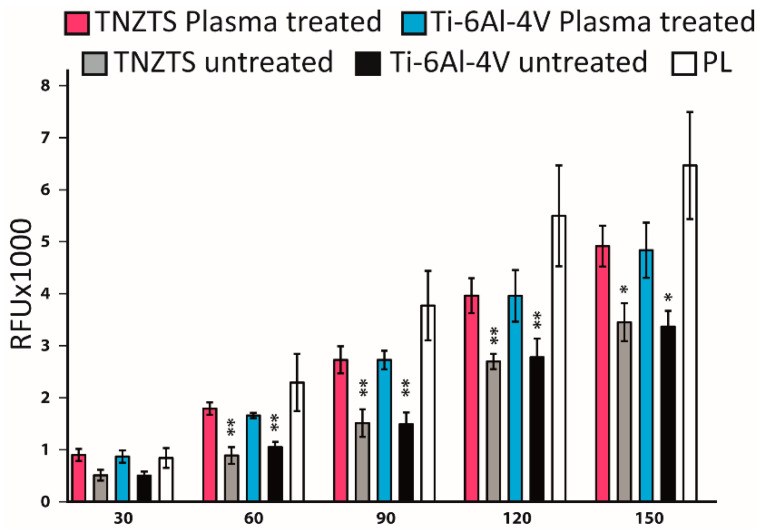
Summary of the results of resazurin reduction in primary human osteoblast cell cultures at 24 h after seeding, indicating mitochondrial activity. Cells were cultured on untreated and oxygen plasma-treated metallic substrates (TNZTS alloy, Ti-6Al-4V) and cell culture plate plastic. The measurements were made over time with 30 min interval (30 min–150 min), data represent mean ± SD; *n* = 3. * for *p* < 0.1; ** for *p* < 0.05 are shown relative to those obtained on cell culture plate plastic.

Primary human osteoblasts showed similar attachment to the TNZTS alloy and Ti-6Al-4V surfaces, with slightly better attachment to the former surface (Figure 12A–D). Furthermore, for each of the alloys, there was a significant increase in the number of viable cells attached to the scaffold surface following O_2_ plasma treatment (Figure 12E–H). Moreover, the mitochondrial activity of osteoblasts grown on untreated substrates was significantly lower than the data recorded for cell culture plate plastic, which was not the case for cells cultured on O_2_-plasma-treated substrates (Figure 13). Data on protein content analysis in cells grown on the same substrates are shown in Figure S2.

### 2.8. The Response of Human PBMCs to Tested Scaffolds

**Figure 14:**
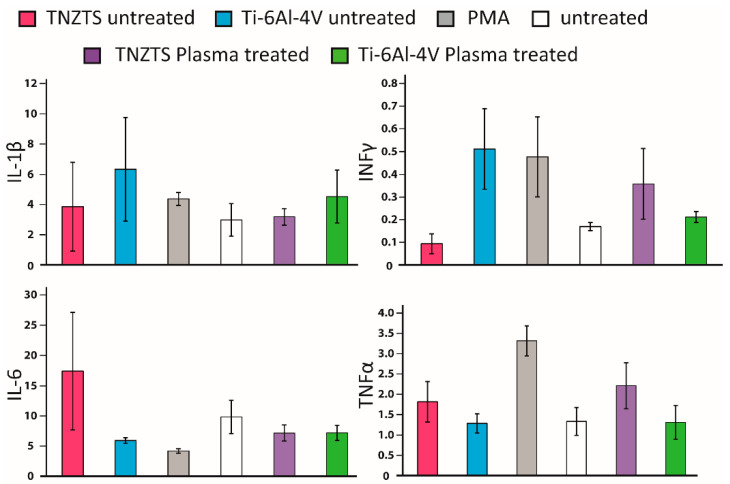
Summary of the results for secretion of cytokines by PBMC-derived macrophages cultured on all tested substrates; that is, TNZTS alloy, Ti-6Al-4V, cell culture plate plastic with PMA inducer (positive control), and cell culture plate plastic (untreated cells, negative control). Data represent mean ± SD; *n* = 3.

The levels of pro-inflammatory proteins were not significantly elevated in O_2_-plasma-treated metallic substrates in comparison to untreated cells (Figure 14), indicating that these substrates would not trigger inflammatory responses by activating macrophages.

## 3. Discussion

Though much progress has been made in the development of biomedical implants, about 20% of patients still suffer from severe problems caused by design, manufacturing, and material selection issues [42]. One material selection issue results in stress shielding, this being the large difference in elastic modulus between the implant alloy (in most cases, the Ti-6Al-4V alloy because of its biocompatibility and low density) and that of the surrounding bone, resulting in bone resorption and, eventually, loosening of the implant [37,42,43,44]. Thus, there is much research interest in the development of low elastic modulus Ti-based alloys [15,42]. Low elastic modulus can be achieved by modification of the composition of the alloy through the addition of certain elements. In the present work, we synthesized and performed a detailed characterization of one such alloy, namely, Ti-38Nb-5Zr-2Ta-2Sn (the TNZTS alloy).

EDX analysis of the local distribution of alloying elements in different areas of the as-cast TNZTS alloy demonstrated a good correlation of the distribution of alloying elements in separate volumes and binary phase diagrams Ti-Nb, Ti-Ta, Ti-Zr, and Ti-Sn [45,46,47]. According to binary phase diagrams, the melting point of Ti-based alloys in comparison with pure Ti increases following the alloying with Nb and Ta but, on the contrary, is reduced when the alloy contains Zr and Sn. Hence, during the solidification of the alloys of Ti-Nb and Ti-Ta systems, the first crystals are enriched with Nb and Ta in relation to the content of the liquid phase. On the other hand, the first crystals of Ti-Zr and Ti-Sn systems are depleted in Zr and Sn. These phenomena were confirmed in our quinary system. In the observed alloy areas, with the elevation of Nb and Ta content, there was a decrease in Zr and Sn and vice versa. In fact, in the course of solidification, the behavior of the alloying elements in TNZTS alloy is consistent with the principles governing the solidification of binary alloys in the Ti-Nb, Ti-Zr, Ti-Ta, and Ti-Sn systems.

The diffraction pattern of the as-cast TNZTS alloy showed non-typical intensity or even the absence of specific peaks of the β-solid solution, which were reported previously [48]. We attribute this to the coarse grain structure of the alloy. It is to be noted that the diffraction pattern was taken from a limited number of β-grains, which, being at a scattering position according to Wulff–Bragg’s condition, may lack certain planes of a β-solid solution.

In the forged state, lattice spacing a_β_ is equal to 0.3296 nm, which is comparable to that of the as-cast alloy (a_β_ = 0.3928 nm). The structure of the forged TNZTS alloy is characterized by significantly finer β grains in comparison to the as-cast alloy (Figure 4a), with its approximately uniform, polyhedric shape and mean size of 200 µm. This is due to recrystallization processes during the thermo-mechanical processing of the bar (forging + annealing) that was used to obtain rod specimens. XRD data (Figure 5) pointed to the differences of preferred orientations of the matrix β-solid solution in longitudinal and transverse directions of the hot-forged rod, presumably contributing to the anisotropy of the elastic modulus; that is, the difference between the mean values of contact moduli were within a range of 0–4 GPa. Lower values of contact modulus, shown for longitudinal direction (60 GPa), are explained by the maximum intensity of the low modulus <110> orientation component [34,49]. However, 4 GPa difference in the mean values of contact moduli in longitudinal and transverse directions is relatively small, as the intensity of the low modulus <110> orientation component is accompanied by the intensity of the high modulus <211> orientation component. 

According to XRD analysis, lattice spacing (a_β_) of the β-phase in the as-cast TNZTS was 0.3298 nm, which is higher than that of a_β_ of pure Ti, extrapolated to room temperature (0.3282 nm [31]). This is due to the doping of the alloy with elements (Nb, Zr, Ta, Sn), each of which has an atomic radius that is greater than that of Ti in β-state (Table 1). Similar values of lattice spacing (a_β_) of beta-phase TNZTS (0.329–0.300 nm) were obtained in biocompatible alloys of Ti-Nb-Ta-Zr [9], Ti-Ta-Sn, and Ti-Ta-Zr [8] systems.

The stress–strain curve of hot forged TNZTS alloy demonstrated the near-absence of a work-hardening effect (Figure 6). Such phenomenon was also observed while conducting a tensile test of the other β-phase, Ti-based biocompatible alloys [50]. This is linked to a so-called dislocation-free plastic deformation mechanism and the formation of the nanometer-scale strain domains during cold deformation. The mechanical and elasticity properties of the TNZTS alloy, recorded with the help of a tensile test (Table 2), are typical for Ti-Nb-system-biocompatible alloys with a metastable β-structure [51].

We attribute the formation of developed polygonal structure in the hot-worked and annealed alloy to high stacking fault energy, typical for β-matrix alloys with bcc crystal structure [52]. Such alloys are characterized by easy cross-gliding of screw dislocations, leading to their faster annihilation and the development of stable polygonal dislocation walls. 

There are two key aspects of the structural and mechanical properties of TNZTS alloy. First, in the as-cast state, the alloy has a single-phase, non-homogenous, large grain structure, with a mean grain size >1 mm. Second, following forging and annealing within the β-region, there is the formation of a recrystallized polyendric structure with a relatively low density of dislocations with a mean grain size of ~200 µm, with the collection of physical and mechanical properties being YS = 515 MPa; UTS = 540 MPa; ε = 12%; RA = 57%; E = 61 ±1 GPa; and HV = 217 ± 4 kg/mm^2^.

In the in vitro cell culture assay studies, fewer SCP-1 and Saos-2 cells attached to the TNZTS alloy substrate than to cell culture plate plastic. A similar trend was observed in the mitochondrial activity and protein content test results. No significant differences in the mitochondrial activity of cells cultured on TNZTS and Ti-6Al-4V were recorded. These results are in agreement with those reported in many studies on the biocompatibility of Ti-based alloys [26,53,54]. Mishchenko et al. [53] found that the values of resazurin reduction obtained for human osteoblasts cultured on commercially pure (cp) Ti specimens were not significantly different from those for Zr-Ti-Nb alloy specimens, at each time point, including 24 h. Similarly, employing MC3T3-E1 pre-osteoblasts, cytotoxicity data of 24 h cell culture did not differ significantly between cells grown on cp Ti and Ti-Nb alloys, with Nb contents of 5, 10, 15, 20, and 25 wt./wt.% [26]. Falanga et al. [54] showed a similar rate of short-term proliferation and viability of human fibroblast cultured on specimens of Ti-based alloys, including a Ti-Nb alloy.

In order to improve cell attachment, we increased the surface wettability of tested substrates by subjecting them to radio-frequency glow discharge O_2_ plasma treatment, which led to a significant increase in the hydrophilicity of both scaffolds (Figure 11). 

Using primary human osteoblasts, we observed a pronounced elevation of cell viability and cell adhesion in comparison with untreated metallic scaffolds (Figure 12 and Figure 13) that was consistent with analogous research on plasma-treated scaffolds [55]. For example, employing fibroblast cell cultures, cell attachment and viability were significantly higher in O_2_-plasma-treated samples, composed of Ti or Zr, in comparison with untreated ones [56]. Furthermore, Wang et al. revealed more efficient cell adhesion, proliferation, and osteocalcin secretion of rat osteoblasts cultured on titanium samples, which underwent a combined Ar-O_2_ plasma treatment [57]. Similar outcomes were recorded for mouse osteoblasts grown on O_2_-Ar-plasma-treated titanium substrates [58]. Thus, O_2_-Ar plasma treatment might represent an alternative to the O_2_ plasma treatment used here when it comes to increasing the hydrophilicity of the TNZTS alloy.

Additionally, in vivo experiments with O_2_-plasma-treated Ti specimens demonstrated enhanced bone healing [59]. Hence, the present results showing significantly increased biocompatibility of O_2_-plasma-treated TNZTS and Ti-6Al-4V alloy scaffolds seeded with primary human osteoblasts contributes to the body of evidence that indicates the excellent potential of O_2_-plasma-treated biomedical Ti-based alloys.

The present research was mainly focused on the physical and mechanical properties (strength, ductility, elastic modulus) and biocompatibility characteristics of TNZTS. Fatigue life, fatigue crack, and corrosion resistance have not been tested. These tests, however, could be included in experimental design modeling exploitation conditions of a specific implant construction. Further research on TNZTS alloy could also include biocompatibility tests in vivo. 

## 4. Materials and Methods

### 4.1. Design of the Ti-38Nb-5Zr-2Ta-2Sn (wt.%) Alloy 

The design of novel compositionally complex Ti-Nb-Zr-Ta-Sn alloy is based on d-electrons concept [60]. To accomplish this, the electron concentration e/a, i.e., the number of valence electrons per atom, as well as [Mo]eq, were determined [32]. The latter characterizes the stability of β-solid solution. According to d-electrons concept [1], Bo and Md parameters were calculated. Bo (bond order) characterizes covalent bond strength between Ti and alloying elements. Md is the metal d-orbital energy level, which correlates the electronegativity and the metallic radius of elements. Bo and Md values are calculated based on the rule of additivity.

Bo = ∑C*_i_* × Bo*_i_*; Md = ∑C*_i_* × Md*_i_*

where C*_i_*—concentration of *i*-alloying element in atomic percent; Bo, Md—calculated for *i*-alloying element.

According to [61], the minimal elastic modulus of β-phase-based Ti-alloys is observed to be close to β/β + ω boundary in the Bo-Md phase stability diagram. In this case, the electron concentration e/a is ≥4.20 [62]. In addition, as was mentioned in Introduction, the minimal value of the Ti-Nb alloy elastic modulus corresponds to [Mo]eq > 11, i.e., 40–42% of Nb concentration. 

In the Ti-Nb system, alloying with β-stabilizers and neutral strengtheners leads to additional reduction of elastic modulus [17,63]. For example, in the Ti-40Nb-xSn system, addition of 2 wt./wt.% Sn results in an elastic modulus of 55 GPa [63]. In low elastic modulus Ti-Nb-Sn alloys, the Sn content does not exceed 4 wt./wt.% [64,65]. In contrast, the Zr content of low elastic modulus Ti-Nb-Ta-Zr alloys (55–60 GPa) is in the range of 4.5–7.0 wt./wt.% [66,67,68], an example, Ti-29Nb-13Ta-4.6Zr ([Mo]eq = 10.7) [3,69,70]. A shortcoming of this alloy is its relatively high cost due to the high content of Ta. Thus, Ti-Nb-Ta-Zr alloys with low Ta content (2 wt./wt.%), such as Ti-36Nb-2Ta-3Zr-0.3O and Ti-35Nb-2Ta-3Zr, have been the subject of a number of studies [3]. However, although these alloys have low elastic modulus (~60 GPa), their Nb content is high (35–36 wt./wt.%) and Zr content is low (0–3 wt./wt.%), resulting in low [Mo]eq (10.2–10.5).

Based on the above-mentioned analysis and the calculations of Bo, Md, e/a, [Mo]eq experimental alloy Ti-38Nb-2Ta-5Zr-2Sn (TNTZS) having the following parameters: Bo = 2.876; Md = 2.455; e/a = 4.26; [Mo]eq = 11 has been proposed. This alloy completely meets the following criteria: Bo–Md lies within the area of metastable β-phase existence, close to β/β + ω phase boundary, e/a ≥ 4.20, [Mo]eq ≥ 11.

### 4.2. Synthesis of the Ti-38Nb-5Zr-2Ta-2Sn (wt.%) Alloy

Blended pure Ti, Nb, Zr, Ta, and Sn powders were used to obtain an ingot of Ti-38Nb-5Zr-2Ta-2Sn (wt./wt.%) alloy (TNZTS alloy). The size distribution of the powders lay in the range of 5–45 µm with d50 = 25 µm. Electrolytic 99.1% pure Ti powder was manufactured at the JSC Corporation VSMPO AVISMA (Verkhnyaya Salda, Sverdlovsk Region, Russia). A 99.5% trace Zr basis powder was produced employing calciumthermic reduction. Nb powder (99.5% purity) was obtained by the aluminothermic process at Ulba Metallurgical Plant (Ust-Kamenogorsk, Republic of Kazakhstan) according to Russian GOST 26252-84. Ta powder (99.5% purity) was manufactured using sodium reduction at Ulba Metallurgical Plant (Ust-Kamenogorsk, Republic of Kazakhstan). Sn powder (99.1% purity) was gas atomized according to GOST 9723-73. To achieve equal distribution of metallic powder granules of the different metals in the mechanical mixture as well as to avoid fractionation, the following steps were undertaken. First, powders with close fraction size (10–40 µm) were mixed; second, the powder of a metal to be used in lower quantity was mechanically mixed with the powder of a metal of higher quantity in an equal ratio, and only after proper mixing the remaining amount of metal that had to be used in higher quantity was gradually added to the mixture. All the metal powders were mixed following this procedure.

The mechanical mixture was pressed by uniaxial compression under force of 100 kN and vacuum of 10^−1^ Pa at the temperature of 950 °C for 15 min. The obtained sintered cylinder billets (diameter = 80 mm, thickness = 10 mm) underwent triple vacuum arc remelting (VAR), conducted at the JSC Corporation VSMPO AVISMA (Verkhnyaya Salda, Sverdlovsk Region, Russia), that yielded a 15 kg cylindrical ingot, with a diameter of 20 cm and a height of 20 cm) (this alloy is hereafter referred to as the as-cast alloy).

**Figure 15:**
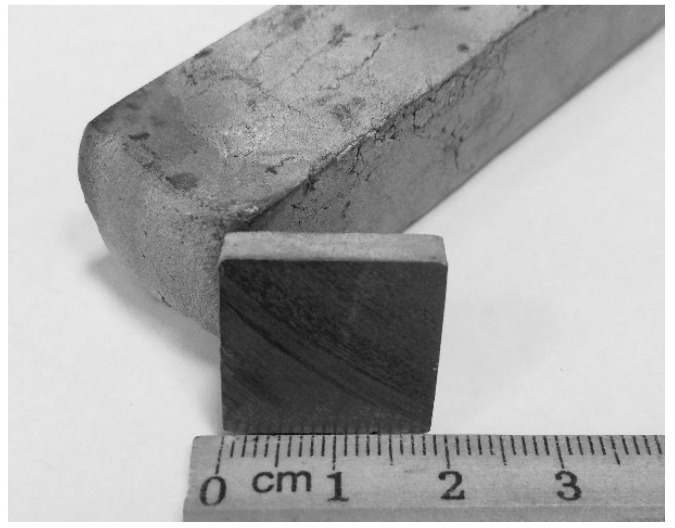
Shape and appearance of TNZTS rod following second forging.

Two-stage hammer forging of the as-cast ingot under heating up to 1050 °C was carried out at the Institute of Metal Physics of the Ural Branch of Russian Academy of Sciences (Yekaterinburg, Russia). The first stage was conducted using a 30 kg hammer, resulting in a rod of intermediate size. The second stage was performed using an 8 kg hammer to obtain a rod of square cross-section with a side length of 19 mm (Figure 15). Forging was conducted with rotation at 90°, and the temperature at each stage was not less than 700 °C. Following the second forging stage, the alloy was heated at 1050 °C and air-quenched inside a sand mold (this alloy is hereafter referred to as the forged alloy).

### 4.3. Scanning Electron Microscopy (SEM)

SEM was conducted using a ZEISS CrossBeam AURIGA scanning electron microscope and electron back-scatter diffraction (EBSD) equipped with Oxford Instruments Inca Energy 250 for EDX analysis (Carl Zeiss NTS, Oberkochen, Germany); in total, 12 images were analyzed.

### 4.4. Transmission Electron Microscopy (TEM)

A total of 16 TEM foils were studied using JEM2100 (Jeol, Tokio, Japan) with an accelerating voltage of 200 kV. Billets for TEM studies were separated using an Ecocut EDM machine (Electronica, Saswad, India). Then, billets were ground with abrasive silicon carbide paper and electrically polished in an electrolyte (10:6:1 volume ratio of methanol:butanol:perchloric acid) at 50 °C.

### 4.5. X-ray Diffraction (XRD)

XRD analysis was carried out using a Bruker D8 Advance diffractometer (Bruker, Mannheim, Germany) and a position-sensitive detector in filtered Cu K-α radiation in the range of Bragg’s angle (2Θ) between 34° and 100°, with a step size of 0.05°. XRD data were analyzed by the Rietveld method [71] using TOPAS software (Bruker, Germany). To conduct thermal XRD, a camera (Anton Paar HTK 1200N (Anton Paar, Austria) was attached.

### 4.6. Dynamic Mechanical Analysis (DMA)

Dynamic mechanical analysis (DMA) was conducted with a DMA 242 C set-up (NETZSCH, Selb, Germany) using specimens sized 25 mm × 4 mm × 1 mm, with the long side being parallel to the direction of the rod axis. Dynamic storage modulus (E*) was determined according to the scheme of three-point bending in an argon atmosphere at maximum dynamic force on sample = 5.3 N, constant quasi-static force = 0.05 N, frequency = 1 Hz. It is pointed out that, based on DMA method, dynamic storage modulus E* was determined not in the direction of applied stress, but in the perpendicular direction, because in such a mode, extension/contracting stress appears along the plate sample axis.

### 4.7. Vickers Hardness, Relaxed Elastic Modulus (E_r_), and Uniaxial Tension Tests

Vickers hardness (HV) and relaxed elastic modulus (Er) of specimens cut across the rod axis were obtained from microindentation tests using CSM ConScan (CSM Instruments, Peuseux, Switzerland) according to the Oliver–Pharr method [72] at a load of 9 N. For each specimen, 12 measurements were made. Uniaxial tension tests on specimens (gage length of 50 mm) cut along the rod axis were conducted using an electromechanical materials testing machine (Instron 3382; Instron, High Wycombe, UK) equipped with an extensometer (at a crosshead displacement rate of 5 mm/min).

### 4.8. Substrate Preparation for Biocompatibility Assays

Circular (diameter = 9 mm) and square (4 mm × 4 mm) specimens were cut from TNZTS alloy (thickness = 0.05 mm). Same-sized Ti-6Al-4V specimens were used as positive control. Before use, specimens were washed thoroughly in tap water and sonicated in isopropyl alcohol for about 30 min.

To obtain hydrophilic surfaces, TNZTS alloy and Ti-6Al-4V specimens were subjected to O_2_ plasma treatment. Specimens, placed in glass Petri dishes, were treated in a low-pressure Dentaplas PC O_2_ plasma system (Diener Electronic, Ebhausen, Germany). After generation of 0.3 mbar (30 Pa) vacuum within 2 min, 160 W plasma was ignited at an oxygen flow of 3 sccm and applied for 15 min at 40 °C.

### 4.9. Isolation of PBMCs and Neutrophil Cells

Blood samples (EDTA blood) were obtained intravenously from 4 healthy volunteers (without implants or health issues) after institutionally approved informed consent (Ethics vote number: 046/2020BO2, University Tübingen). Peripheral blood mononuclear cells (PBMCs) and neutrophils were isolated by Lympholyte-poly (Cedarlane, Burlington, Canada) gradient centrifugation, as described previously [73]. Briefly, 6 mL of EDTA blood were layered carefully over 6 mL of Lympholyte-poly separation medium in a 15 mL centrifuge tube (three tubes were used per donor). After centrifugation at 500× *g* for 35 min at 22 °C (no brakes), layers of PBMCs and neutrophils were collected into 15 mL Falcon tubes, diluted with phosphate-buffered saline (PBS) (about 12 mL of PBS per tube), and centrifuged at 400× *g* for 10 min at 22 °C. This washing step was repeated once. Finally, peripheral blood mononuclear cells (PBMCs) and neutrophils were resuspended in 2 mL of RPMI-1640 medium (±phenol red) containing 2% autologous plasma (Merck, Darmstadt, Germany). For ELISA, cell density of PBMCs was 0.9 × 10^6^/mL.

### 4.10. Cell Culture

A human immortalized bone-marrow-derived mesenchymal stem cell line (SCP-1 cells) was cultured in α-MEM (minimal essential medium; GIBCO, Carlsbad, USA) containing 1 g/L glucose supplemented with 5% FBS (fetal bovine serum; GIBCO, Germany) [74]. Osteogenic Saos-2 and suspension THP-1 cell lines obtained from DSMZ (Leibniz-Institut-Deutsche Sammlung für Mikroorganismen und Zellkulturen GmbH) were cultured in RPMI-1640 medium (5% FCS). These cell lines were used for no longer than 15 passages. Human primary osteoblasts [75] were isolated from donor samples (BG Clinic, Tübingen) in accordance with the Ethics vote of the University Hospital Tübingen (539/2016BO2) after obtaining the patient’s written consent. Briefly, cancellous bone was removed mechanically from the bone tissue and washed with PBS. Following 1 h collagenase digestion (0.07% collagenase II in PBS) at 37 °C, released osteoblasts were transferred to cell culture flasks in culture medium (DMEM, 5% FCS, 1% penicillin/streptomycin, 50 µM L-ascorbate-2 phosphate, 50 µM β-glycerol phosphate) for expansion. Medium was changed every 4–5 days. Experiments were performed in passages 3 or 4. All cells were cultured in a humidified incubator (37 °C and 5% CO_2_) [75]. For cell culture assays, the following cell densities were used: 0.8 × 10^5^/mL (SCP-1), 1.2 × 10^5^/mL (Saos-2, THP-1).

### 4.11. Enzyme-Linked Immunosorbent Assay (ELISA)

The levels of cytokines (that is, tumor necrosis factor alpha (TNF-α), interleukin 1 beta (IL-1β), interleukin 6 (IL-6), and interferon gamma (IFN-γ)), in 24 h PBMC culture supernatants were quantified using standard ABTS ELISA kits (900-K25, 900-K95, 900-K16, 900 K-27—PeproTech, Hamburg, Germany). All ELISAs were conducted according to the manufacturer’s protocols.

### 4.12. Fluorometric Quantification of NET-DNA Release

The 0.5 × 10^5^ neutrophils were seeded into each well of a 48 well plate. Medium contained 1 µM cell impermeable SYTOX Green (Thermo Fisher Scientific, Darmstadt, Germany) to stain released DNA. Positive control included cells stimulated with PMA (phorbol 12-myristate 13-acetate/100 nM). For normalization, cells were lysed with Trition X-100 (0.5%). The fluorescence of NET-bound SYTOX Green (excitation λ: 488 nm, emission λ: 510 nm) was measured every 30 min for a period of 4.5 h at 37 °C using a FLUOstar^®^ Omega multi-mode microplate reader (BMG Labtech, Offenburg, Germany) in fluorescence units (RFU) that reflect the free DNA concentration [73].

### 4.13. Resazurin Assay

Cell proliferation was evaluated using resazurin conversion assay [76]. Briefly, cells were incubated with 1:10 volume of sterile resazurin (Sigma, Darmstadt, Germany) working solution (0.025% in PBS) at 37 °C. Resulting fluorescence (excitation λ: 540 nm, emission λ: 590 nm) was measured using a FLUOstar^®^ Omega multi-mode microplate reader every 30 min up to 150 min. Fluorescence value correlates with the cell number.

### 4.14. Live–Dead Staining

Viable cells were detected at day 1 post-seeding by intracellular esterase activity using Calcein-AM. Briefly, cells were incubated with 2 µM Calcein-AM (Biomol, Hamburg, Germany) and Hoechst 33342 (Sigma, Munich, Germany) at room temperature (protected from light) for 30 min. Following a washing step with PBS, images were taken using an EvosFL epifluorescence microscope (Life Technologies, Darmstadt, Germany).

### 4.15. Protein Quantification

To verify resazurin assay data, cell density on tested substrates (TNZTS, Ti-6Al-4V, cell culture plastic) was analyzed by quantifying the total protein content in all three culture conditions using sulforhodamine B (SRB). Cells were washed once with PBS and fixed with 99.9% ethanol at −20 °C for at least 1 h. Briefly, ethanol was removed, cells were washed with tap water, air-dried, and stained with 0.4% (*w*/*v*) SRB in 1% (*v*/*v*) acetic acid for 30 min. Next, the dye was removed by washing 3–4 times with 1% (*v*/*v*) acetic acid. Bound SRB was resolved in 10 mM Tris-based solution for spectrophotometric measurement at 550 nm using a FLUOstar^®^ Omega multi-mode microplate reader [77].

### 4.16. Cell Morphology

Following washing of cells seeded scaffolds with PBS, cells were fixed using 4% paraformaldehyde for 10 min at RT; then, cells were permeabilized with 0.25% Triton-X solution for 20 min at RT. Next, cells were incubated with 1% BSA (Bovine Serum Albumin, Sigma) blocking solution for 1 h. Then, actin fibers and nuclei were stained with Alexa Fluor 555 Phalloidin (1:500, Santa Cruz Biotechnology, Heidelberg, Germany) and Hoechst 33342, respectively, for 45 min in the dark. Images were captured using a EvosFL epifluorescence microscope.

### 4.17. Sessile Drop Contact Angle Measurement

Wettability of untreated and O_2_-plasma-treated samples was determined by automatic drop application and contact angle evaluation employing a drop shape analysis system (DSA 10-Mk2, Kruess, Hamburg, Germany). Water droplets (2 µL) were deposited on the surfaces of the samples, and drop shape and infiltration behavior were recorded by a video camera (1 frame/s). Contact angles of water droplets, provided that they were not infiltrated, were measured 20 s after the initial surface contact by drop shape analysis with a circular segment method (circle fitting) implemented in Kruess software (Kruess, Germany).

### 4.18. Statistical Analysis

Quantitative results are presented as mean ± standard deviation. Test of significance of difference between population means was conducted using analysis of variance (ANOVA) followed by two-tailed unpaired Student’s *t*-test (Anderson–Darling normality test was performed); *p* < 0.05 was considered statistically significant.

## 5. Conclusions

For the novel Ti-based alloy that we designed and synthesized (TNZTS alloy), we found (i) an elastic modulus that is significantly lower than that of Ti-6Al-4V; (ii) biocompatibility measures that were comparable to corresponding ones for Ti-6Al-4V; (iii) significantly increased hydrophilicity after O_2_ plasma treatment, resulting in increased cell adhesion and cell viability; and (iv) negligible release of tested cytokines. This collection of properties suggests that the TNZTS alloy has good potential to be used as an alternative to Ti-6Al-4V to fabricate implantable medical devices, such as dental posts and total joint replacements.

## Figures and Tables

**Table 1:** A selection of properties of titanium, zirconium, tantalum, niobium, and tin [15,16].

Property/Metal	Ti	Zr	Nb	Sn	Ta
Concentration in Earth’s crust (ppm) [16]	5600	190	20	2.2	2
Atomic mass (g/mol)	47.90	91.22	92.91	118.69	180.95
Atomic radius (pm) [29]	146.15/142.11 *	160.25/155.36 *	142.9	162	143
Density at room temperature (kg/m^3^) [16]	4.540	6.506	8.570	7.310	16.654
Elastic modulus (GPa) [29]	116	68	105	50	186

* The second value is the calculated atomic radius (r) at room temperature for high-temperature bcc lattice metal modification (β-Ti, β-Zr) [r = (√3)a]/4], where a is the period of bcc lattice extrapolated for room temperature, which considers thermal expansion. For β-Ti, a = 328.2 pm [30] and for β-Zr, a = 358.78 pm [31].

**Table 2:** Summary of mechanical properties of forged TNZTS alloy compared to certified Ti-based alloys used in medicine.

Alloy Designation	YS (MPa)	UTS (MPa)	EL (%)	RA (%)	HVμ	E (GPa)	E* (GPa)	E_r_ (GPa)
TNZTS	515 ± 5	540 ± 5	12 ± 1	57 ± 2	217 ± 4	63 ± 3	60 ± 2	62 ± 2
CP-Ti, grade 1 [37]	170	240	24	-	-	115	-	-
CP-Ti, grade 4 [38]	530	620	19	-	220	115	-	-
Ti-6Al-4V ELI, annealed [38]	895	930	16	-	310	114	-	-
Ti-6Al-7Nb, hot-rolled and annealed [39]	880–950	1050	20	-	325	114	-	-
Ti-13Nb-13Zr [37]	840–910	970–1040	10–16	-	-	79–84	-	-
Ti-15Mo [37]	655	800	22	-	-	78	-	-

YS—yield strength; UTS—ultimate tensile strength; EL—elongation at break; RA—reduction of area; HVμ—Vickers microhardness; E—modulus of elasticity; E*—dynamic storage modulus; E_r_—relaxed elastic modulus.

## Supplementary Materials

The following are available online at https://www.mdpi.com/article/10.3390/ijms221910611/s1.
