# Supplementary material for: Synthesis and Characterization of a Novel Biocompatible Alloy, Ti-Nb-Zr-Ta-Sn
Source: Int J Mol Sci. 2021 Sep 30;22(19):10611. doi: 10.3390/ijms221910611 (PMC8509052; doi:10.3390/ijms221910611)
Supplement: Supplementary file 1 [file ijms-22-10611-s001.zip › ijms-1380894-supplementary.pdf]

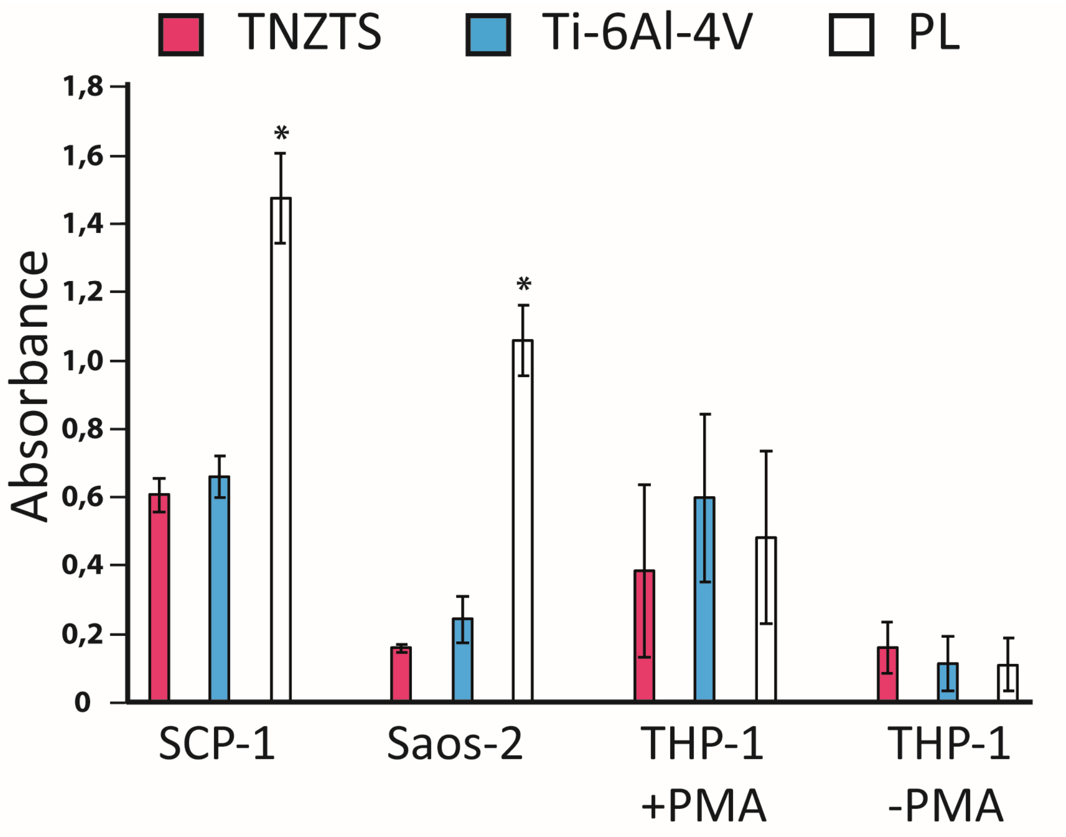

Figure S1: SRB absorbance indicating protein content which is indirect measurement of cell proliferation. SRB measurements were conducted for SCP-1, Saos-2, THP-1 (with and without PMA inducer) cultured on TNZTS, Ti-6Al-4V and cell culture plate plastic (PL) for 24 h. * for $p<0.1$.

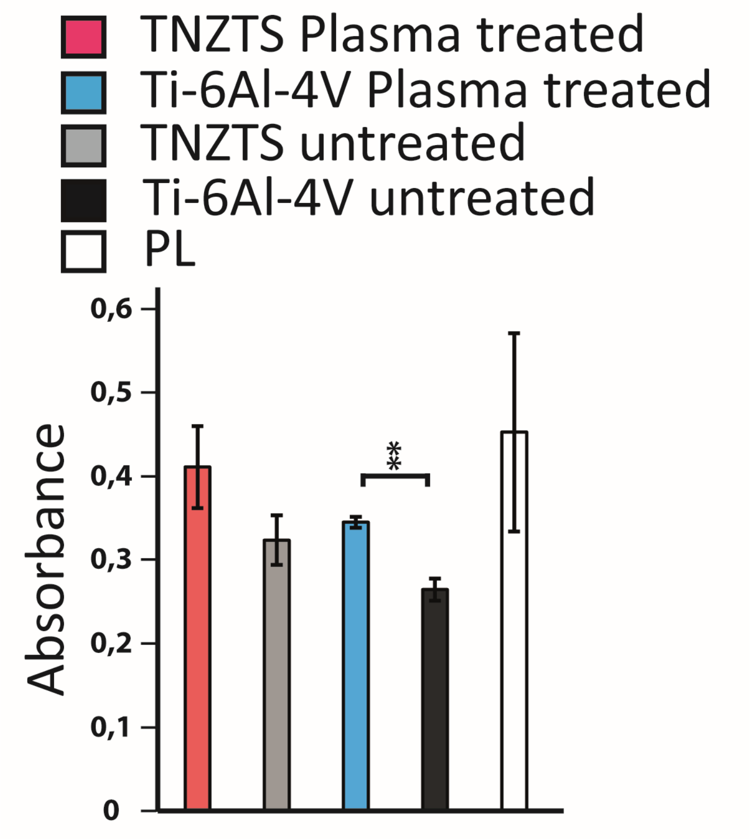

Figure S2: SRB absorbance indicating protein content which is indirect measurement of cell proliferation. SRB measurements were conducted for primary human osteoblasts cultured on untreated and oxygen plasma treated metallic substrates (Ti-6Al-4V and TNZTS) and control (cell culture plate plastic) for 24 h. ** for p<0.05.
